# Comprehensive Receptor Repertoire and Functional Analysis of Peripheral NK Cells in Soft Tissue Sarcoma Patients

**DOI:** 10.3390/cancers17152508

**Published:** 2025-07-30

**Authors:** Luana Madalena Sousa, Jani-Sofia Almeida, Tânia Fortes-Andrade, Patrícia Couceiro, Joana Rodrigues, Rúben Fonseca, Manuel Santos-Rosa, Paulo Freitas-Tavares, José Manuel Casanova, Paulo Rodrigues-Santos

**Affiliations:** 1 Laboratory of Immunology and Oncology, Center for Neuroscience and Cell Biology (CNC), University of Coimbra, 3004-504 Coimbra, Portugal; lmossousa@gmail.com (L.M.S.); janisofiaalmeida@hotmail.com (J.-S.A.); claretef.acs@gmail.com (T.F.-A.); patycouceiro@gmail.com (P.C.); 2 Center for Innovation in Biomedicine and Biotechnology (CIBB), University of Coimbra, 3000-504 Coimbra, Portugal; msrosa@fmed.uc.pt (M.S.-R.); jmpscasanova@gmail.com (J.M.C.); 3 Institute of Immunology, Faculty of Medicine (FMUC), University of Coimbra, 3000-504 Coimbra, Portugal; 4 Center of Investigation in Environment, Genetics and Oncobiology (CIMAGO), Faculty of Medicine, University of Coimbra, 3000-548 Coimbra, Portugal; 5 Coimbra Institute for Clinical and Biomedical Research (iCBR), Faculty of Medicine, University of Coimbra, 3000-548 Coimbra, Portugal; 6 Clinical Academic Centre of Coimbra (CACC), 3004-561 Coimbra, Portugal; rodrigues.joana.mc@gmail.com (J.R.); ruben_fonseca@msn.com (R.F.); pftavares@gmail.com (P.F.-T.); 7 Tumor Unit of the Locomotor Apparatus (UTAL), Orthopedics Service, University Clinic of Orthopedics, Coimbra Local Health Unit (ULSC), 3000-075 Coimbra, Portugal

**Keywords:** soft tissue sarcoma, natural killer cells, innate immunity, innate lymphoid cells, receptor repertoire, cancer immunotherapy

## Abstract

Background: Soft tissue sarcomas (STSs) are a rare and heterogeneous group of mesenchymal tumors with limited response to current therapies, particularly in advanced stages. STS tumors were traditionally considered “cold” tumors, characterized by limited immune infiltration and low immunogenicity. However, emerging evidence is challenging this perception, highlighting a potentially critical role for the immune system in STS biology. Objective: Building on our previous findings suggesting impaired natural killer (NK) cell activity in STS patients, we aimed to perform an in-depth characterization of peripheral NK cells in STS. Methods: Peripheral blood samples from STS patients and sex- and age-matched healthy donors were analyzed to assess NK cell degranulation, IFNγ production, and receptor repertoire. Results: Functional assays revealed a notable reduction in both degranulation and IFNγ production in NK cells from STS patients. STS patients also exhibited dysregulated expression of activating and inhibitory NK cell receptors. Principal component analysis (PCA) identified CD27 and NKp44 as critical markers for distinguishing STS patients from healthy donors. Increased CD27 expression represents a shift towards a more regulatory NK cell phenotype, and we found that CD27 expression was negatively correlated with NK cell degranulation and IFNγ production. ROC curve analysis demonstrated strong potential to distinguish between the groups for both CD27 (AUC = 0.85) and NKp44 (AUC = 0.94). Conclusion: In conclusion, STS patients exhibited impaired NK cell function, altered receptor repertoire, and a shift towards a less cytotoxic and more regulatory phenotype.

## Simple Summary

Soft tissue sarcomas (STSs) are rare cancers that respond poorly to current treatments. As the immune system is increasingly recognized as a key player in cancer control, we investigate a specific type of immune cell—natural killer (NK) cells. We analyzed NK cells from blood samples of STS patients and healthy individuals. NK cells from STS patients were less functional, showing reduced degranulation and lower production of IFNγ, both essential for killing cancer cells. We also found alterations in key surface markers, which may explain their impaired function. Among these, CD27 and NKp44 were notably different between patients and healthy individuals and showed strong potential to distinguish between these groups. Higher CD27 levels were associated with NK cells that are less effective at killing cancer cells. Overall, our findings suggest that NK cells from STS patients may have a reduced capacity to fight cancer.

## 1. Introduction

Soft tissue sarcomas (STSs) are a heterogeneous and rare group of malignant tumors, with over 100 different histopathologic subtypes identified and representing only 1% of solid tumors [1,2,3]. Standard STS therapy includes surgery, with or without radiotherapy, and chemotherapy [1]. The rarity and heterogeneity of STS pose significant challenges for accurate diagnosis. Furthermore, the complexity of the disease often renders existing treatments ineffective for many patients, leading to poor survival rates and a high incidence of recurrence and metastasis [4]. Consequently, there is an urgent need for novel therapeutic strategies and biomarkers to improve patient outcomes and better forecast the course of disease.

The great advances of immunotherapy have revolutionized the treatment of several solid tumors. Interestingly, while the history of immunotherapy began with Coley’s work on sarcomas, the development of immunotherapeutic strategies for STS stagnated for decades [5]. STS tumors were traditionally considered “cold” tumors, characterized by limited immune infiltration. However, emerging evidence is challenging this perception, highlighting the immune system’s critical role in STS [6,7,8,9]. Therefore, understanding the intricate interactions between the immune system and STS may unveil novel disease biomarkers and therapeutic targets, ultimately advancing patient care and outcomes.

The immune contexture in STS tumors is characterized by notable features, including a dysfunction of tumor-infiltrating lymphocytes (TILs), particularly reduced NK cell activity [10]. While understanding the tumor’s local immune microenvironment is critical, systemic immunity also plays a pivotal role in cancer control, influencing and reflecting tumor responses [11]. Despite the advantages of easier sample accessibility and the potential for longitudinal patient monitoring, research focusing on systemic immunity in STS remains limited. With this in mind, in previous work, we performed deep immunophenotyping and gene expression analysis of peripheral blood from STS patients [12]. Among other conclusions, this study suggested the compromised cytotoxic potential associated with NK cells, evidenced by a decreased frequency of cytotoxic NK cells and reduced gene expression of cytotoxic granules, such as PRF1 and GZMB, and KLRK1, which encodes for the activating receptor NKG2D. These results indicate that disruptions in the phenotype and functionality of NK cells could play a pivotal role in STS progression, highlighting the need for further investigation into their contributions to this disease.

Unlike T cells, which rely on antigen-specific receptors, NK cells utilize a diverse array of non-antigen-specific receptors to distinguish between self- and non-self-targets [13,14]. Commonly, NK cells are subdivided into two major subpopulations, CD56^bright^ NK cells, with higher potential for cytokine production, and CD56^dim^ NK cells, with greater cytotoxic potential [15]. Numerous NK cell receptors have been identified to date, including the CD94/NKG2 receptor family, which encompasses inhibitory receptors like NKG2A and activating receptors such as NKG2C and NKG2D, as well as the natural cytotoxicity receptors (NCRs), which include the receptors NKp30, NKp44, and NKp46, involved in the recognition of tumor cells. Since these receptors play a critical role in regulating NK cell function, studying their repertoire has been instrumental in elucidating NK cell behavior in several cancers [16,17,18].

In this context, the present study aimed to deeply characterize peripheral NK cells in STS patients to better understand their function and phenotype. Our findings reveal impaired NK cell function and altered receptor repertoire, pointing to a less cytotoxic profile that may contribute to a limited immune response against these tumors.

## 2. Materials and Methods

### 2.1. Study Design

The patient demographic data and clinicopathological characterization is presented in Table 1. We included primary, recurrent, metastatic, and recurrent/metastatic patients with different histologies, tumor locations, and chemotherapy treatments. No treatment-naïve patients were included. From these patients, peripheral blood samples were collected at the Orthopedic Service from Coimbra Local Health Unit (ULSC), with previous signed informed consent to participate in this study. A control group of 21 healthy donors (CTRL), with a median age of 66 years and 60% female, was also included. Buffy coats from the CTRL were collected at the Blood and Transplantation Center of Coimbra of the Portuguese Institute for Blood and Transplantation (IPST). The present study was approved by the Ethical Committees of the Faculty of Medicine of the University of Coimbra (CE-167/2020, CE-018/2021) and the ULSC, Portugal (CHUC-021-19).

### 2.2. NK Cell Degranulation and Intracellular Staining of IFNγ

Peripheral blood mononuclear cells (PBMCs) were isolated from peripheral blood using a standard density gradient protocol (Lymphoprep™, STEMCELL Technologies, Grenoble, France). PBMCs and the K562 (ATCC CCL-243) cell line (American Type Culture Collection, Manassas, VA, USA) were cultured in an effector-to-target ratio of 25:1 for 4 h at 37 °C in RPMI 1640 medium (Life Technologies, Paisley, UK). Immediately before culture, the PE-conjugated monoclonal antibody anti-CD107a and 10 μg/mL brefeldin A (Sigma, Saint Louis, MO, USA) were added to block the secretion of proteins to the extracellular space. Detailed antibody specifications are provided in Table S1. After incubation, the cells were washed and resuspended in 100 μL of PBS, followed by staining with membrane monoclonal antibodies anti-CD56 and anti-CD3. Stained cells were incubated for 15 min at room temperature in the dark. Next, cells were treated with Fix and Perm A solution (Invitrogen, Carlsbad, CA, USA) and incubated for 10 min. After centrifugation at 450× *g* for 5 min, the supernatant was discarded, and Fix and Perm B solution (Invitrogen, Carlsbad, CA, USA) along with intracellular monoclonal antibody anti-IFNγ were added. Following incubation, cells were centrifugated, washed, and resuspended in PBS before acquisition on an 8-color BD FACSCanto II cytometer (Becton Dickinson, San Jose, CA, USA).

### 2.3. Flow Cytometry Analysis

Peripheral blood samples, 100 μL or up to 1 × 10^6^ leukocytes, were incubated with fluorochrome-conjugated monoclonal antibodies for 15 min in the dark at room temperature. The antibody panel employed enabled the relative quantification of 23 extracellular key receptors related to activation, inhibition, and maturation on total NK cells, and its CD56^bright^ and CD56^dim^ NK cell subsets. Detailed antibody specifications are provided in Table S1. After staining, erythrocytes were lysed using 2 mL of BD lysing solution (BD Biosciences, San Jose, CA, USA) for 10 min in the dark at room temperature. Then, the cell suspensions were centrifuged at 450x *g* for 5 min, and the supernatants were discarded. The pellets were washed once with phosphate-buffered saline (PBS) buffer and acquired in an 8-color flow cytometer, BD FACSCanto II (BD Biosciences, San Jose, CA, USA). Data analysis was performed using FlowJo v.10.7 (BD Biosciences, San Jose, CA, USA), and the gating strategy is represented in Figure S1.

### 2.4. Statistical Analysis

For data visualization, radar plots were created using Python (version 3.8.2). Normalized median values are represented with shaded areas. For comparisons between groups, correlation between variables, principal component analysis (PCA), and ROC curve analysis, GraphPad Prism 9.2.0 for macOS (GraphPad Software, San Diego, CA, USA) was used. The normalization of flow cytometry data was performed with arcsin transformation. Comparisons of the variables between STS patients and CTRL were performed with Mann–Whitney U test. Significance levels were set at *p* < 0.05. The data is presented as the median ± inter-quartile range (IQR) of the original values. Spearman’s correlation coefficient was calculated to evaluate the correlation between the receptor’s expression and the expression of CD107a and IFNγ. Significance levels were also set at *p* < 0.05. For the principal component analysis, normalized data were scaled, and missing values were imputed using the median. ROC curve analysis was performed to assess the performance of NK cell receptor expression levels in distinguishing STS patients from CTRL, and the area under the curve (AUC) was calculated.

## 3. Results

In line with our previous findings that STS patients might have compromised NK activity due to the decrease in the frequency of the circulating most effector subset CD56^dim^ NK cells and the decrease in the expression of PRF1, GZMB, and KLRK1 [12], we performed a more detailed characterization of NK cytotoxic activity and receptor repertoire. This assessment was conducted in 12 STS patients and 21 CTRL peripheral blood samples by flow cytometry.

Despite the smaller sample size, we observed a consistent reduction in the absolute frequency of total NK cells in the peripheral blood of STS patients (0.1 × 10^3^ cells/μL, IQR: 0.08–0.2, *N* = 11) compared with CTRL (0.2 × 10^3^ cells/μL, IQR: 0.1–0.4, *N* = 21, *p* = 0.01) (Figure S2). Additionally, the previously reported decrease in the frequency of CD56^dim^ NK cells was still observed in our cohort of STS patients (87% of NK, IQR: 82–91, *N* = 12) compared with CTRL (95% of NK cells, IQR: 93–98, *N* = 21, *p* = 0.0006).

### 3.1. Impaired Degranulation and IFNγ Production Highlight NK Cell Dysfunction in STS Patients

**Figure 1:**
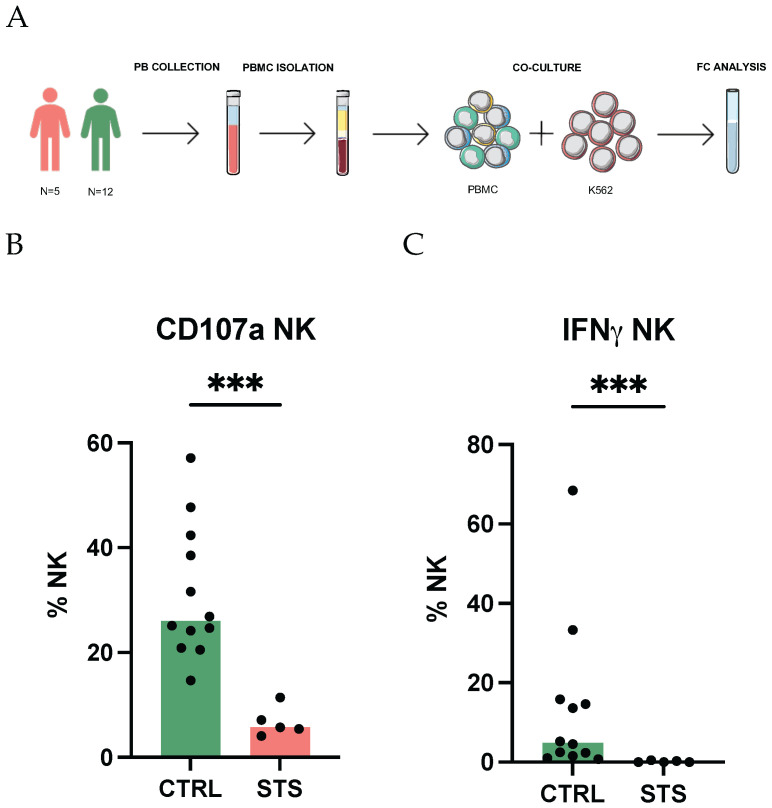
Impairment of IFNγ production by NK cells. (**A**) Schematical representation of the methodology used to assess NK cell degranulation and cytotoxicity. PBMCs, isolated from peripheral whole blood samples, were co-cultured with K562 cells, stained with intracellular and extracellular antibodies, and analyzed by flow cytometry. Total NK cells were identified as CD3− CD56+. (**B**) Relative frequency of total NK expressing CD107a observed in STS patients and CTRL. (**C**) Relative frequency of total NK expressing IFNγ observed in STS patients and CTRL. Arcsin-transformed values were used for statistical analysis, and differences between groups were assessed using Mann–Whitney U tests. Significant differences between STS patients and healthy donors are marked with asterisks (*p*-value < 0.05 *, <0.01 **, <0.001 ***, <0.0001 ****). Legend: PB, peripheral blood; PBMC, peripheral blood mononuclear cell; FC, flow cytometry; NK, natural killer; IFNγ, interferon gamma; CTRL, healthy donors control group; STS, soft tissue sarcoma group.

To characterize the NK cell compartment in STS, we evaluated the functional capacity of these cells by assessing their ability to degranulate and produce IFNγ upon co-culture with K562 target cells (Figure 1A). Degranulation was assessed indirectly by measuring the surface expression of CD107a, and IFNγ production was evaluated via intracellular staining.

Our results revealed that the degranulation capacity of NK cells was compromised in STS patients. Specifically, the frequency of CD107a-expressing NK cells after K562 stimulation significantly decreased in STS patients (6%, IQR: 5–7, *N* = 5) compared with CTRL (26%, IQR: 23–39, *N* = 12, *p* = 0.0003) (Figure 1B). Furthermore, a significant reduction in the frequency of IFNγ-producing NK cells was observed in STS patients (0%, IQR: 0–0.3, *N* = 5) compared with CTRL (5%, IQR: 2–15, *N* = 12, *p* = 0.0003), indicating a weakened pro-inflammatory response (Figure 1C).

### 3.2. Comprehensive Phenotypic Profiling Reveals Altered NK Cell Receptor Repertoire in STS Patients

Since we observed an impaired degranulation ability and IFNγ production, we performed a detailed phenotypic characterization of NK cells, and a comprehensive panel of receptors was assessed via flow cytometry. These receptors included (1) activating receptors (CRACC, DNAM-1, NKG2C, NKG2D, NKp30, NKp44, NKp46, and NKp80), which enhance NK cell function and cytotoxicity; (2) inhibitory receptors (CD96, LAG-3, NKG2A, PD-1, TIGIT, and TIM-3), which regulate NK cell activity, preventing overactivation; (3) adhesion and differentiation markers (CD11b, CD27, CD57, and CD62L), which are associated with NK cell maturation and migration; and (4) functional activation markers (CD69, CD137, CD137L, and HLA-DR), which reflect the activation status of NK cells.

**Figure 2:**
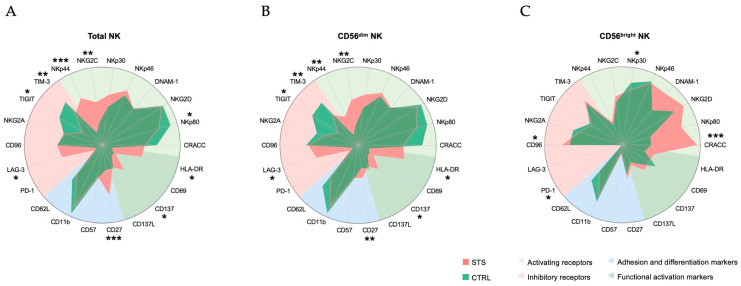
Global alteration of NK receptor repertoire. Peripheral whole blood samples were stained with extracellular antibodies and analyzed by flow cytometry. Radar plots show the arcsin-normalized relative frequency of NK cells expressing the different receptors, with each receptor represented on an individual axis with a specific scale. Receptors are grouped by function: activating receptors (light green sector), inhibitory receptors (red sector), adhesion and differentiation markers (blue sector), and activation markers (dark green sector). The shaded red area of the radar plot represents the median normalized values for STS patients, and the shaded green area represents the median normalized values for the healthy donors. (**A**) Total NK cells were identified as CD3−CD56+. (**B**) The subpopulations CD56^bright^ were discriminated based on high CD56 expression. (**C**) The subpopulations CD56^dim^ were discriminated based on low CD56 expression. Arcsin-transformed values were used for statistical analysis, and differences between groups were assessed using Mann–Whitney U tests. Significant differences between STS patients and healthy donors are marked with asterisks (*p*-value < 0.05 *, <0.01 **, <0.001 ***, <0.0001 ****).

The comparison of the relative frequency of total NK cells expressing each receptor between STS patients and CTRL revealed significant alterations (Figure 2A). Total NK cells expressing activating receptors, particularly NKG2C and NKp44, were increased in the peripheral blood of STS patients (16%, IQR: 10–23, *N* = 10; 9%, IQR: 4–13, *N* = 6; respectively) compared with CTRL (5%, IQR: 4–10, *N* = 21, *p* = 0.003; 1%, IQR: 1–2, *N* = 21, *p* = 0.0003; respectively). This suggests that NK cells in STS patients may have been exposed to activating stimuli. In contrast, total NK cells expressing NKp80, a co-stimulatory receptor associated with mature NK cells, were decreased in STS patients (99%, IQR: 97–99, *N* = 6) compared with CTRL (99%, IQR: 99–100, *N* = 21, *p* = 0.047). This downregulation may indicate a shift towards a less mature NK cell phenotype in STS. Among inhibitory receptors, the frequency of total NK cells expressing LAG-3 was increased in STS patients (2%, IQR: 0.4–3, *N* = 7) compared with CTRL (0.07%, IQR: 0.03–0.15, *N* = 11, *p* = 0.01), possibly reflecting a state of functional exhaustion. Conversely, TIGIT- and TIM-3-positive NK cells were decreased in STS patients (43%, IQR: 30–54, *N* = 12; 48%, IQR: 40–49, *N* = 7; respectively) compared with CTRL (66%, IQR: 56–71, *N* = 21, *p* = 0.01; 67%, IQR: 58–79, *N* = 11, *p* = 0.006; respectively). Additionally, NK cells expressing the differentiation-related marker CD27 expression were increased in STS patients (11%, IQR: 9–13, *N* = 12) compared with CTRL (5%, IQR: 3–9, *N* = 21, *p* = 0.0005). This upregulation may indicate a skewing toward a less mature NK phenotype. Finally, total NK cells positive for functional activation-related receptors, particularly CD137 and HLA-DR, were also significantly increased in STS patients (1%, IQR: 1–2, *N* = 12; 11%, IQR: 3–20, *N* = 12; respectively) compared with CTRL (0.4%, IQR: 0.3–0.8, *N* = 21, *p* = 0.02; 2%, IQR: 1–3, *N* = 21, *p* = 0.02; respectively), once again indicating exposure to activating stimuli.

Given the functional heterogeneity of NK cells, we assessed the expression of the same receptors within the CD56^bright^ and CD56^dim^ NK subsets, which have distinct roles in cancer immunity. Most alterations observed in total NK cells were also present in CD56^dim^ NK cells, except for the reduction in the frequency of CD56^dim^ NK cells expressing NKp80, which did not reach significance (Figure 2B). In the CD56^bright^ NK cells, specific alterations in their receptor repertoire between STS patients and CTRL were noted (Figure 2C). The frequency of CD56^bright^ NK cells expressing the activating receptor CRACC was increased, while NKp30-positive CD56^bright^ were decreased in STS patients (100%, IQR: 99–100, *N* = 6; 84%, IQR: 70–95, *N* = 12; respectively) compared with CTRL (79%, IQR: 73–84, *N* = 11, *p* = 0.0002; 94%, IQR: 89–98, *N* = 21, *p* = 0.03; respectively). Furthermore, the frequency of CD56^bright^ NK expressing the inhibitory receptors CD96 and PD-1 was increased in STS patients (91%, IQR: 89–95, *N* = 12; 0.0%, IQR: 0.0–0.3, *N* = 11; respectively) compared with CTRL (81%, IQR: 75–89, *N* = 21, *p* = 0.04; 0.0%, IQR: 0.0–0.0, *N* = 11, *p* = 0.04; respectively).

### 3.3. Principal Component Analysis Identifies CD27 and NKp44 as Key Discriminators of NK Cell Alterations in STS Patients

**Figure 3:**
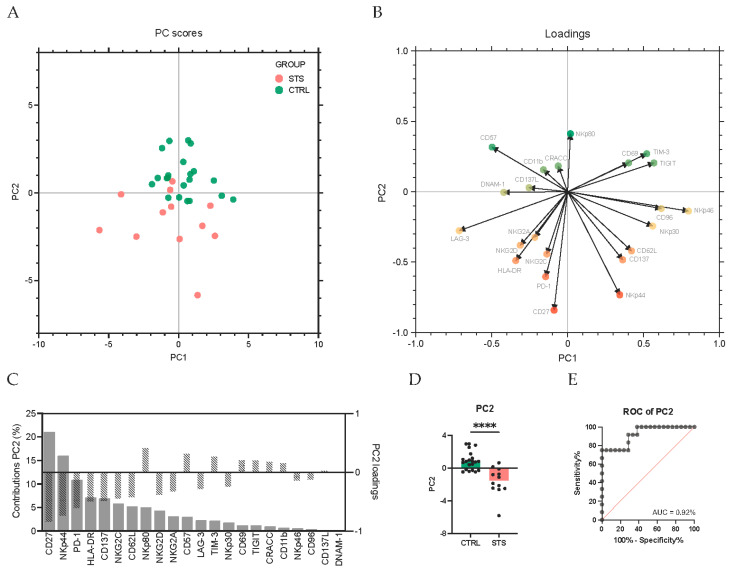
Principal component analysis highlights CD27 and NKp44 as key markers. Peripheral whole blood samples were stained with extracellular antibodies and analyzed by flow cytometry. Total NK cells were identified as CD3−CD56+. Principal component analysis (PCA) of the arcsin-normalized values of the frequency of total NK cells expressing the different receptors was conducted. PCA was performed using standardized center data, and missing values were imputed using the median of the respective receptor across all samples. Single value decomposition (SVD) was used to compute the PC. (**A**) The PCA plot illustrates the distribution of STS patients (red dots) and the CTRL group (green dots). (**B**) Loading plot illustrating the contribution of each NK receptor to the variability captured by PC1 and PC2 in the PCA analysis. (**C**) Bar plot representing the contributions and loadings of individual NK receptor to PC2 in the PCA analysis. The left *y*-axis (gray bars) represents the percentage of contribution of each receptor to PC2. The right *y*-axis (striped bars) represents the loading values for each receptor on PC2. Receptors with positive loadings contribute positively to the corresponding principal component, whereas receptors with negative loadings contribute negatively. (**D**) PC2 scores for STS patients and CTRL. The differences between groups were assessed using Mann–Whitney U tests. Significant differences between STS patients and healthy donors are marked with asterisks (*p*-value < 0.0001 ****). (**E**) ROC curve of PC2 scores differentiating STS patients from CTRL. Legend: CTRL, healthy donors control group; STS, soft tissue sarcoma group; PC, principal component; ROC, receiver operating characteristic; AUC, area under the curve.

Having first conducted univariate analyses on the frequencies of NK cell expressing each receptor, where no clear trend in the direction of activating or inhibition was observed, we next aimed to understand whether these differences reflected a broader shift in the overall NK cell phenotype. To address this, we performed a PCA integrating the expression of all NK cell receptors. PCA is an unsupervised multivariate method that reduces dimensionality and identifies patterns in the data. This approach enabled us to determine whether NK cell phenotypes naturally cluster between groups. This analysis showed that, although principal component 1 (PC1) captures the largest proportion of total variance, PC2 was the one that most clearly separated STS patients from CTRL (Figure 3A). Most STS patients exhibited negative PC2 scores, while the CTRL group tended to have elevated and positive PC2 scores. Indeed, the PC2 scores are significantly lower in STS patients (−1.5, IQR: −2.5–−0.6, *N* = 12) compared to CTRL (0.8, IQR: −0.2–1.2, *N* = 21, *p* < 0.0001) (Figure 3D), and the ROC curve analysis for PC2 revealed an AUC of 0.92, indicating excellent discriminatory power (Figure 3E).

To determine which receptors contributed most significantly to group segregation, we analyzed the contribution of each receptor to PC2 and identified CD27 and NKp44 as the receptors with the highest contributions (21% and 16% contribution to PC2, respectively), both with negative loadings (−0.8 and −0.7, respectively), suggesting that increased expression of these markers is associated with lower PC2 scores and hence with the STS group (Figure 3B,C).

**Figure 4:**
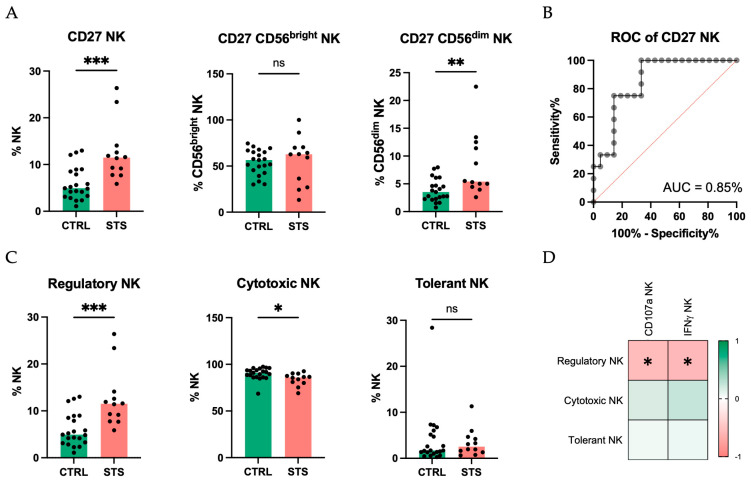
CD27: A marker of regulatory NK cell expansion and cytotoxic decline. Peripheral whole blood samples were stained with extracellular antibodies and analyzed by flow cytometry. Total NK cells were identified as CD3−CD56+, and the subpopulations CD56^bright^ and CD56^dim^ were discriminated based on high and low CD56 expression, respectively. (**A**) Relative frequency of total NK, CD56^bright^, and CD56^dim^ NK subsets expressing CD27 observed in STS patients and CTRL. (**B**) ROC curve of frequency of NK cells expressing CD27 differentiating STS patients from CTRL. (**C**) Relative frequency of regulatory (CD27+CD11b+/−), cytotoxic (CD27−CD11b+), and tolerant (CD27−CD11b−) NK cells observed in STS patients and CTRL. Differences between groups were assessed using Mann–Whitney U tests. (**D**) Correlation analysis of regulatory, cytotoxic, and tolerant NK cells, with the frequency of NK cells expressing CD107a and IFNγ. Spearman’s correlation analysis was performed. The color scale represents the direction of association; green means positive correlation, and red means negative correlation. Significant differences are marked with asterisks (*p*-value < 0.05 *, <0.01 **, <0.001 ***, <0.0001 ****). Legend: CTRL, healthy donors control group; STS, soft tissue sarcoma group; ROC, receiver operating characteristic; AUC, area under the curve.

Given its significant contribution to PC2, which differentiates STS patients from the CTRL group, we focused our analysis on the expression of CD27. A significant increase in the frequency of NK cells expressing CD27 was observed in STS patients (11%, IQR: 9–13, *N* = 12) compared with the CTRL group (5%, IQR: 3–9, *N* = 21, *p* = 0.0005) (Figure 4A). This upregulation was also evident in the CD56^dim^ NK subset but not in the CD56^bright^ subset from STS patients (5%, IQR: 5–12, *N* = 12; 63%, IQR: 34–70, *N* = 7; respectively) compared with the CTRL (4%, IQR: 2–5, *N* = 21, *p* = 0.004; 57%, IQR: 46–65, *N* = 21, *p* = 0.6; respectively). In addition, to evaluate the discriminative potential of CD27, we performed a ROC curve analysis, which yielded an AUC of 0.85 (Figure 4B), underscoring its potential as a biomarker for STS.

Further, using CD27 and CD11b expression, NK cells can be classified into three distinct subsets: regulatory (CD27+CD11b+/−, cytotoxic (CD27−CD11b+), and tolerant (CD27−CD11b−). In STS patients, we observed an increase in regulatory NK cells and a decrease in cytotoxic NK cells (11%, IQR: 9–13, *N* = 12; 86%, IQR: 81–88, *N* = 12; respectively) compared with CTRL (5%, IQR: 3–9, *N* = 21, *p* = 0.0005; 91%, IQR: 86–94, *N* = 21, *p* = 0.01; respectively) (Figure 4C).

Finally, to understand the functional implications of these alterations in STS patients, we correlated the frequency of these subgroups with the percentage of NK cells capable of degranulation and IFNγ production. Regulatory NK cells showed a significant negative correlation with both the frequency of CD107a-positive NK cells (Spearman r = −0.6, *p* = 0.04) and the frequency of NK cells producing IFNγ (Spearman r = −0.6, *p* = 0.02) (Figure 4D).

**Figure 5:**
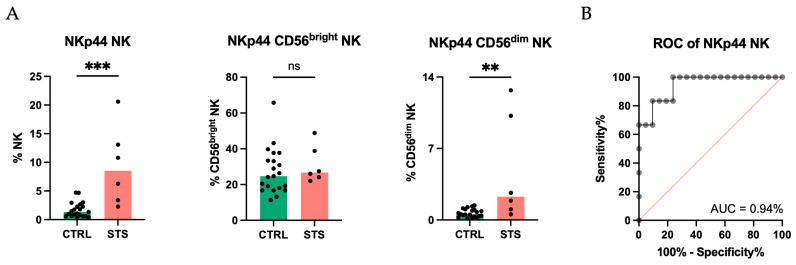
NKp44: elevated expression with high diagnostic potential. Peripheral whole blood samples were stained with extracellular antibodies and analyzed by flow cytometry. Total NK cells were identified as CD3− CD56+, and the subpopulations CD56^bright^ and CD56^dim^ were discriminated based on high and low CD56 expression, respectively. (**A**) Relative frequency of total NK, CD56^bright^, and CD56^dim^ NK subsets expressing NKp44 observed in STS patients and CTRL. Differences between groups were assessed using Mann–Whitney U tests. (**B**) ROC curve of frequency of NK cells expressing NKp44 differentiating STS patients from CTRL. Significant differences are marked with asterisks (*p*-value < 0.05 *, <0.01 **, <0.001 ***, <0.0001 ****). Legend: CTRL, healthy donors control group; STS, soft tissue sarcoma group; ROC, receiver operating characteristic; AUC, area under the curve.

Similar to CD27, we also focused on NKp44 due to its significant contribution to PC2. A significant increase in the frequency of total NK cells expressing NKp44 in STS patients (9%, IQR: 4–13, *N* = 6) compared with CTRL (1%, IQR: 1–2, *N* = 21, *p* = 0.0003) (Figure 5A) was recorded. This increase was also observed within the CD56^dim^ subset of STS patients but was not detected in the CD56^bright^ subset (2%, IQR: 1–8, *N* = 6; 27%, IQR: 25–36, *N* = 6; respectively) compared to CTRL (1%, IQR: 0–1, *N* = 21, *p* = 0.004; 25%, IQR: 18–33, *N* = 21, *p* = 0.4; respectively). To determine whether the NKp44 expression in NK cells could have a clinical relevance in distinguishing STS patients, we performed a ROC analysis, which yielded an AUC of 0.94 (Figure 5B), highlighting its strong potential to distinguish STS patients from healthy donors.

## 4. Discussion

Although STSs are traditionally considered “cold tumors”, increasing evidence highlights the immune system’s critical role in these malignancies [9]. However, immunological studies in STS patients remain scarce, presenting an opportunity to explore the immune context of this disease and unravel immune-related biomarkers in this context [19]. Recent studies emphasize the relevance of immune signatures and immune-related markers as potential biomarkers in this disease [20]. While most of these studies focus on the local immune microenvironment, exploring the systemic immune landscape offers a complementary approach, with the advantages of being non-invasive and enabling real-time patient monitoring.

In previous work, we performed deep immunophenotyping and gene expression analysis of whole blood from STS patients and observed a decreased frequency of CD56^dim^ NK cells and reduced expression of PRF1, GZMB, and KLRK1 [12]. These findings suggest impaired NK cell-mediated anti-tumor response in these patients. Importantly, in vitro studies have shown that STS cell lines are susceptible to NK cell-mediated cytotoxicity [21], and, in several sarcoma subtypes, high NK cell infiltration correlates with improved patient survival [22].

Given the potential role of NK cells in STS and our previous findings indicating impaired NK function, we aimed to further characterize circulating NK cells from these patients by assessing their ability to degranulate and produce IFNγ upon stimulation with target cells along with a comprehensive mapping of their receptor repertoire.

### 4.1. Impaired Degranulation and IFNγ Production Highlight NK Cell Dysfunction in STS Patients

NK cells are characterized by their production of IFNγ and large numbers of cytolytic granules, such as perforin and granzyme B [23]. Upon target cell recognition, these granules are released into the immunological synapse, a process marked by the surface expression of CD107a [24,25]. Our findings of impaired NK cell degranulation and IFNγ production in STS patients align with previous studies in other malignancies, such as acute myeloid leukemia and various solid tumors, but contrast with observations in melanoma patients, where degranulation is often preserved [26,27,28]. This functional impairment of NK cells in STS patients could compromise their ability to mount an effective anti-tumor immune response. So, restoring NK cell activity could be a potential therapeutic strategy.

### 4.2. Comprehensive Phenotypic Profiling Reveals Altered NK Cell Receptor Repertoire in STS Patients

The NK cells’ adequate immune response relies on a delicate balance between activating and inhibitory signals, mediated by their receptor repertoire [29]. Our detailed analysis revealed significant differences in circulating NK cell receptor expression between STS patients and CTRL, including activating and inhibitory receptors, as well as differentiation and activation markers. These findings suggest that circulating NK cells in STS patients present an altered receptor repertoire, potentially contributing to the compromised function and preventing an efficient anti-tumor response.

We observed upregulation of HLA-DR and CD137, two markers associated with NK cell activation [30]. While in healthy individuals, the frequency of circulating NK cells expressing these markers are generally low, pathological conditions frequently result in its increased expression [31,32,33,34]. In line with findings in melanoma patients [26], their upregulation in STS patients suggests ongoing immune stimulation. However, activation marker expression alone does not necessarily correlate with functional competence, as evidenced by the impaired degranulation and IFNγ production observed.

The balance between activating and inhibitory receptors is crucial for the activation or inhibition of NK cell cytotoxic function and IFNγ production, respectively.

Contrary to expectations and reports in other cancers [35,36,37], we did not observe an increase in the inhibitory receptor NKG2A-positive NK cells in STS patients. However, we did observe an increased frequency of NK cells expressing the activating receptor NKG2C. Similarly, we found an increased frequency of NK cells expressing the activating NCR NKp44 in STS patients, while studies in peripheral blood of patients with other malignancies, such as colorectal cancer, pancreatic cancer, and gastric cancer, have reported decreased expression of NCR [38,39,40]. In contrast, NKp80, a co-stimulatory molecule and a critical marker of functionally mature NK cells [41,42], showed reduced expression in STS patients. Given its association with enhanced cytotoxicity and IFNγ production, this decrease could explain the impaired functional capacity of these cells.

In cancer, the expression of activatory or inhibitory immune checkpoints is often deregulated, contributing also to the immune resistance [43]. For instance, increased expression of inhibitory checkpoints such as LAG-3, TIGIT, and TIM-3 has been documented in various cancers [44,45,46,47,48,49,50,51,52]. In our study, we observed a significant increase in LAG-3-positive NK cells in STS patients compared to controls, consistent with a suppressed cytotoxic function and a tendency towards exhaustion. In tumor-infiltrating NK cells from non-small-cell lung cancer and melanoma, a similar upregulation of LAG-3 was observed [53,54]. However, contrary to these reports, TIM-3- and TIGIT-positive NK cells were decreased in STS patients. The role of TIM-3 in NK cells remains controversial. Although TIM-3 is traditionally classified as an inhibitory checkpoint, its expression has been associated with both inhibitory and activating roles depending on the ligand involved [55]. Indeed, studies have reported that TIM-3-positive NK cells can exhibit greater cytolytic activity, challenging the conventional classification of TIM-3 as a purely inhibitory molecule [56,57,58].

In summary, our detailed analysis of the NK cell receptor repertoire in STS patients highlights a complex dysregulation in STS patients. While the increased expression of certain activation markers suggests that NK cells in STS patients are exposed to activating stimuli, this effect may be counterbalanced by the increased expression of some inhibitory receptors. Additionally, the reduced frequency of the most cytotoxic NK cell subset and the reduced production of IFNγ further supports the notion that, despite signs of activation, the cytotoxic capacity of NK cells in STS patients may be impaired.

### 4.3. Principal Component Analysis Identifies CD27 and NKp44 as Key Discriminators of NK Cell Alterations in STS Patients

Our multivariate analysis highlighted a distinct NK cell receptor expression profile in STS patients. CD27 and NKp44 were identified as the most prominent receptors contributing to the separation of STS patients from CTRL, both with increased expression in STS patients.

CD27 is a co-stimulatory receptor from the tumor necrosis factor receptor superfamily, typically associated with early stages of NK cell differentiation [59]. Its increased expression in NK cells from STS patients suggests a shift towards a less mature or altered functional state. Interestingly, this increase in CD27 expression was particularly pronounced in the CD56^dim^ subset, traditionally considered the more cytotoxic population of NK cells. No significant changes were detected in the CD56^bright^ subset. Furthermore, recent studies have indicated CD27 as a key marker for distinguishing between NK cell subsets [60,61,62,63]. Our findings align with previous reports that most NK cells in peripheral blood do not express CD27 and thus belong to the cytotoxic subset (CD27−CD11b+) [59]. In healthy donors, approximately 6% of peripheral blood NK cells express CD27 [61]. However, in the peripheral blood of STS patients, we observed an increased frequency of regulatory NK cells (CD27+CD11b+/−) and a decrease in cytotoxic NK cells (CD27−CD11b+). This shift indicates a dysregulated NK cell phenotype in STS patients. A similar reduction in conventional cytotoxic NK cells (CD3− CD16^bright^ CD56^dim^) has been described in the peripheral blood of patients with other malignancies [64]. However, to the best of our knowledge, this is the first study to assess NK subsets based on CD27 and CD11b expression in the peripheral blood of STS patients. Finally, we also observed a negative correlation between the frequency of regulatory NK cells and both the frequency of CD107a-positive NK cells and the frequency of NK cells producing IFNγ, reinforcing the functional impact of this phenotype shift. Although these associations are intriguing, our data are correlative, and further studies are needed to determine whether CD27 directly influences NK cell functionality in STS.

NKp44 is a tumor-recognition NCR implicated in the recognition and killing of several cancers cells and is typically upregulated upon cytokine-mediated activation [65,66,67]. NKp44 is constitutively expressed only on CD56^bright^ NK cells. In agreement with this, we observed that only 0.6% of CD56^dim^ NK cells expressed NKp44 in the peripheral blood of healthy individuals. In contrast, STS patients exhibited a significant increase in the frequency of NK cells expressing NKp44, particularly within the CD56^dim^ subset, possibly reflecting persistent stimulation or activation of NK cells in response to tumor signals and suggesting an altered activation state in these patients. This finding is intriguing as it contradicts data from studies on other malignancies, such as colorectal, pancreatic, and gastric cancers, where a downregulation of NKp44 was reported [38,39,40]. Conversely, in breast cancer, contrasting observations have been reported. While one study found no differences in NKp44 expression between patients and controls [64], another reported a higher expression of NKp44 in CD56^dim^ NK cells from patients compared to healthy individuals, similar to our findings in STS patients [68]. Moreover, in circulating NK cells from melanoma patients, an increased percentage of NK cells expressing NKp44 was observed with the advancement in the cancer stages [26]. Similarly, in DLBCL there was a significant increase in CD56+ NKp44+ after PHA stimulation in patients compared to healthy donors [69].

Finally, the diagnostic potential of CD27 and NKp44 expression was emphasized by the ROC analysis, which yielded an AUC of 0.85 and 0.94, respectively, highlighting the strong potential of CD27 and NKp44 to distinguish STS patients from healthy donors. This result aligns with the growing interest in NK cell receptors as potential biomarkers for cancer diagnosis and prognosis.

### 4.4. Study Limitations

Some limitations of our study must be acknowledged. First, the total cohort size is relatively small, which reflects the rarity of STS. Second, although it is common in STS research to include multiple histological subtypes, a large proportion of our patients were diagnosed with leiomyosarcoma, which may limit the generalizability of our findings across the diverse STS histologies. Importantly, all patients included in our study had received therapy prior to sample collection, including those classified as having primary tumors, meaning that truly treatment-naïve patients were not included in our cohort. Additionally, due to the low incidence of STS and the heterogeneity of treatments, we included patients across different treatment regimens and at various time points in both treatment and disease stage to ensure a sufficient sample size. We recognize that previous therapies and disease stage might influence the NK cell phenotype and function, which is a potential confounding factor in our study. So, further research with larger, more homogenous cohorts, ideally including treatment-naïve patients and focusing on specific histological subtypes, are needed to validate and extend our findings.

## 5. Conclusions

Our study provides a comprehensive characterization of NK cells in STS patients. The observed duality in the expression of activating and inhibitory receptors underscores a complex dysregulation within the NK cell population, without a clear trend favoring either activating or inhibitory receptor. Within all the receptors analyzed, CD27 and NKp44 were identified as key markers distinguishing STS patients from healthy donors, with both receptors exhibiting elevated expression in total and CD56^dim^ NK cells. The increased frequency of NK cells expressing CD27 suggests a shift towards a more regulatory NK cell phenotype, at the expense of a cytotoxic phenotype. Finally, in terms of functional activity, the reduction in degranulation capacity and IFNγ production in STS patients suggests a compromised anti-tumor immune response.

Our findings suggest that NK cells from STS patients exhibit significant alterations in their receptor repertoire and impaired degranulation and IFNγ production. Moreover, we highlight the potential of both CD27 and NKp44 as biomarkers for STS, supported by their strong diagnostic performance in distinguishing patients from controls. Overall, this study underscores the importance of understanding NK cell dynamics in STS to inform the development of more effective immunotherapeutic strategies.

## Figures and Tables

**Table 1:** Demographic and clinicopathological data of the patient cohort.

Clinicopathological Characteristic	Value	Frequency (%)
N	12	
Median age (range), years	57 (19–78)	
Sex		
Female	5	42
Male	7	58
Soft tissue sarcoma histology		
Leiomyosarcoma	5	42
Liposarcoma	2	17
Synovial sarcoma	2	17
Haemangiosarcoma	1	8
Malignant fibrous histiocytoma	1	8
Clear cell sarcoma	1	8
Localization		
Connective and soft tissue of limb	3	25
Retroperitoneum	2	17
Connective and soft tissue of head	1	8
Connective and soft tissue of abdomen	1	8
Connective and soft tissue of trunk	1	8
Connective and soft tissue of pelvis	1	8
Jejunum	1	8
Myometrium	1	8
Adrenal gland	1	8
Tumor type		
Primary	2	17
Recurrent	2	17
Metastatic	4	33
Recurrent/Metastatic	4	33
Therapy		
Anthracycline-based therapy	1	8
Trabectedin-based therapy	2	17
Anthracycline- and trabectedin-based therapy	6	50
Anthracycline- and trabectedin-based therapy and others	3	25

## Data Availability

The data presented in this study are available on request from the corresponding author due to ethical and confidentiality restrictions.

## Supplementary Materials

The following supporting information can be downloaded at: https://www.mdpi.com/article/10.3390/cancers17152508/s1, Table S1: Fluorochrome-conjugated monoclonal antibodies used in flow cytometry analysis; Figure S1: Gating strategy used for flow cytometry data analysis; Figure S2: STS exhibited a decrease in the absolute number of NK cells and absolute and relative frequency of CD56^dim^ NK.

## Abbreviations

The following abbreviations are used in this manuscript:

AUC	Area under the curve
CTRL	Control group
IPST	Portuguese Institute for Blood and Transplantation
IQR	Inter-quartile range
NCR	Natural cytotoxicity receptor
NK	Natural killer
PBMC	Peripheral blood mononuclear cell
PBS	Phosphate-buffered saline
PC2	Second principal component
PCA	Principal component analysis
ROC	Receiver operating characteristic
STS	Soft tissue sarcoma
SVD	Single value decomposition
TIL	Tumor-infiltrating lymphocyte
ULSC	Coimbra Local Health Unit
