# Supplementary material for: Comprehensive Receptor Repertoire and Functional Analysis of Peripheral NK Cells in Soft Tissue Sarcoma Patients
Source: Cancers (Basel). 2025 Jul 30;17(15):2508. doi: 10.3390/cancers17152508 (PMC12346150; doi:10.3390/cancers17152508)
Supplement: Supplementary file 1 [file cancers-17-02508-s001.zip › cancers-3752621-supplementary.pdf]

cancers MDPI

Supplementary Material

Table S1. Fluorochrome-conjugated monoclonal antibodies used in flow cytometry analysis.

Antibody	Conjugate	Clone	Brand	Cat#	RRID
CD3	APC-H7	SK7	BD Pharmingen™	560176	AB_1645475
CD3	V500	UCHT1	BD Horizon™	561416	AB_10612021
CD11b	V450	ICRF44	BD Horizon™	560480	AB_1645555
CD19	APC-H7	SJ2501	BD Pharmingen™	560177	AB_1645470
CD20	APC-H7	2H7	BD Pharmingen™	560734	AB_1727449
CD27	FITC	O323	BioLegend®	302806	AB_314297
CD56	PE	B159	BioLegend®	318306	AB_604101
CD56	PerCP/Cy5.5	HCD56	BD Pharmingen™	560842	AB_2033964
CD57	PB	HNK-1	BioLegend®	359608	AB_2562459
CD62L	FITC	DREG-56	BioLegend®	304804	AB_314464
CD69	FITC	FN50	BioLegend®	310904	AB_314839
CD94	FITC	HP-3D9	BD Pharmingen™	555888	AB_396200
CD96	PE	NK92.39	BioLegend®	335406	AB_2275880
CD107a	PE	H4A3	BD Pharmingen™	555801	AB_396135
CD137	APC	4B4-1	BD Pharmingen™	550890	AB_398477
CD137L	PE	C65-485	BD Pharmingen™	559446	AB_397244
LAG-3	PE	T47530	BD Pharmingen™	565616	AB_2571727
DNAM-1	FITC	TX25	BD Pharmingen™	559788	AB_397329
PD-1	APC	MIH4	BD Pharmingen™	558694	AB_1645458
NKG2D	APC	1D11	BioLegend®	320808	AB_492962
CRACC	PE	162.1	BioLegend®	331806	AB_2239190
NKp46	PE	9E2	BioLegend®	331908	AB_1027666
NKp44	AF647	P44-8	BioLegend®	325112	AB_2149431
NKp30	AF647	P30-15	BioLegend®	325212	AB_2149448
HLA-DR	FITC	G46-6	BD Pharmingen™	555811	AB_396145
HLA-DR	V500	G46-6	BD Horizon™	561224	AB_10563765
IFNγ	V450	B27	BD Horizon™	560371	AB_1645594
NKG2A	PE	37006	R&D	FAB1059P	AB_2132978
NKG2C	APC	134591	R&D	FAB138A	AB_416838
NKp80	PE	5D12	BioLegend®	346706	AB_1967147
TIGIT	APC	741182	R&D	FAB7898A	AB_3652758
TIM-3	AF647	7D3	BD Pharmingen™	565558	AB_2744367

***Legend**: FITC - Fluorescein isothiocyanate, PE - R-phycoerythrin, PerCP-Cy5.5 - peridinin chlorophyll protein-cyanine 5.5, PE-Cy7 - R-phycoerythrin – cyanine 7, APC – Allophycocyanin, AF647 – Alexa Fluor 647, APC-H7 – Allophycocyanin cyanine H7, V450 - Violet 450, PB – Pacific Blue 3-carboxy-6,8-difluoro-7-hydroxycoumarin, V500 – Violet 500.*

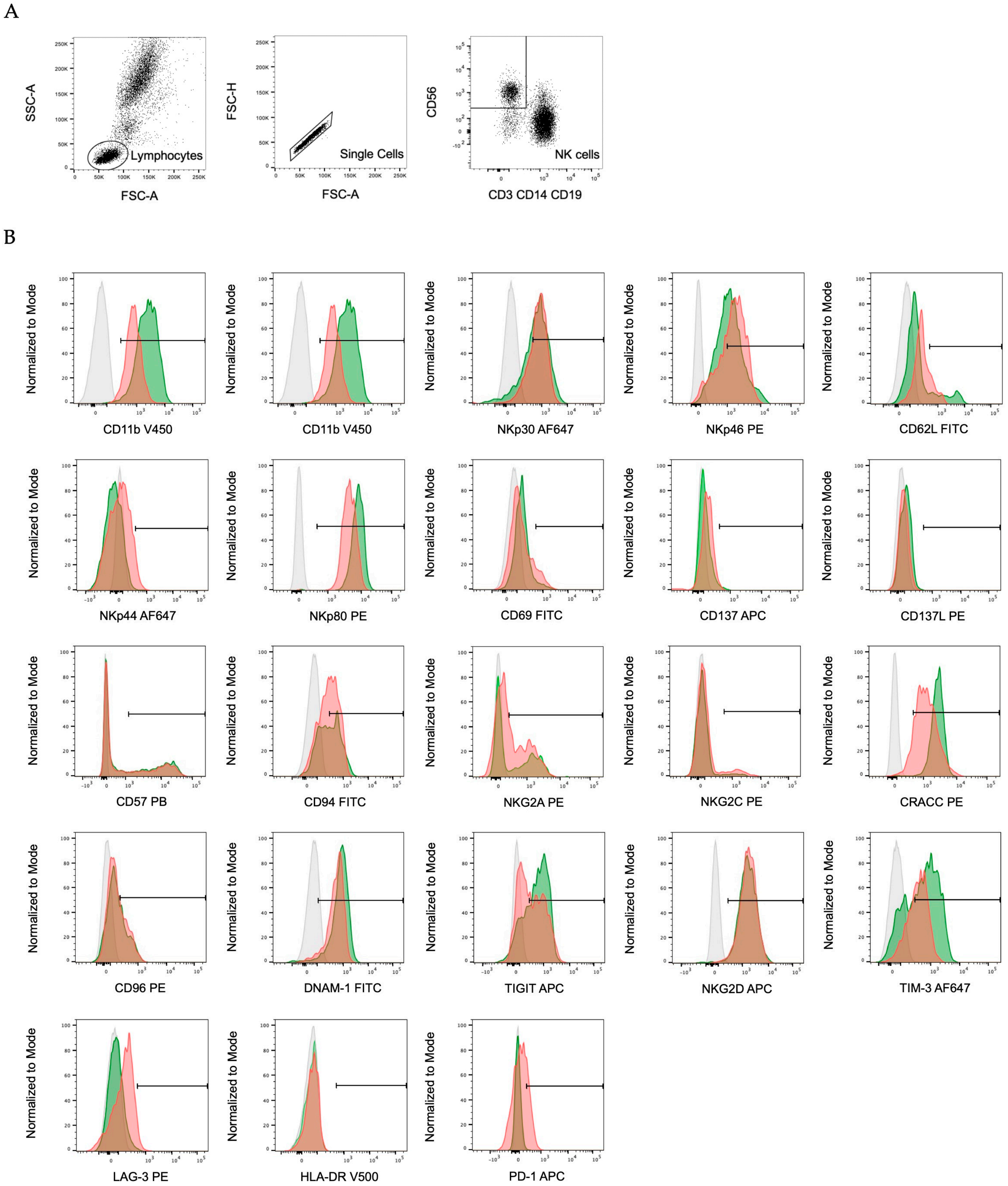

Figure S1: Gating strategy used for flow cytometry data analysis. (**A**) Gate strategy to identify NK cells (CD3$^-$CD56$^+$) and their subpopulations. In the first dot plot the lymphocyte population is defined and, in the second, the single cells are gated. In the third, NK cells (CD3$^-$CD56$^+$) and their subpopulations CD56^{bright} and CD56^{dim} are discriminated. (**B**) Representative histograms relative to surface receptors expression by NK cells. The fullfilled grey lines represent the isotype control, the fulfilled green lines represent the CTRL group, and the fulfilled red lines represent the STS group.

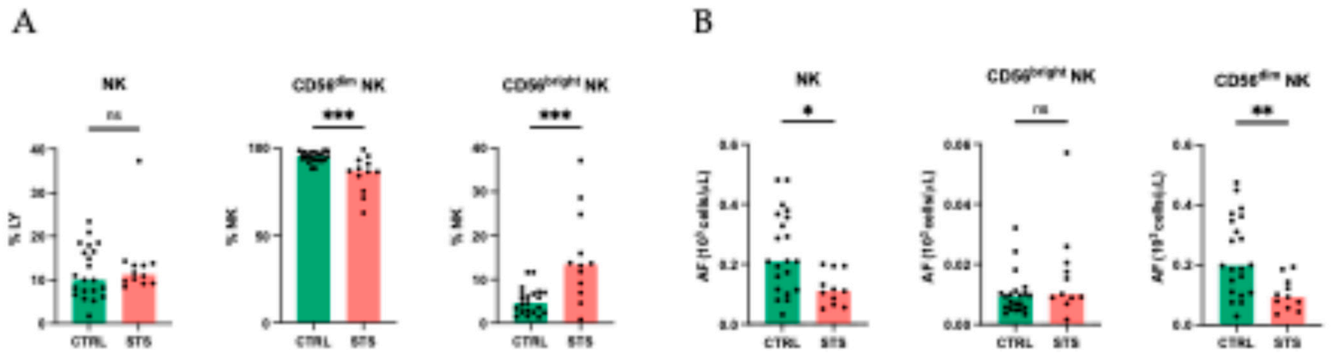

Figure S2: STS exhibited decreased of absolute number of NK cells and absolute and relative frequency of $CD56^{dim}$ NK cells. Peripheral whole blood samples were stained with extracellular antibodies and analyzed by flow cytometry. Total NK cells were identified as CD3- CD56+, and the subpopulations $CD56^{bright}$ and $CD56^{dim}$ were discriminated based on high and low CD56 expression, respectively. (**A**) Relative frequency of total NK, $CD56^{bright}$, and $CD56^{dim}$ NK subsets observed in STS patients and CTRL. (**B**) Absolute frequency of total NK, $CD56^{bright}$, and $CD56^{dim}$ NK subsets observed in STS patients and CTRL. Differences between groups were assessed using Mann-Whitney U tests. Significant differences are marked with asterisks (p-value < 0.05*, < 0.01**, < 0.001***, <0.0001****). Legend: LY, lymphocytes; NK, natural killer; AF, absolute frequency; CTRL, healthy donors control group; STS, soft tissue sarcoma group.
